# O-GlcNAcylation: A bridge for regulating the function of the “heart-kidney-bone axis”

**DOI:** 10.1016/j.bbrep.2026.102635

**Published:** 2026-05-15

**Authors:** Shuangcui Wang, Maojuan Guo, Bo Yang, Tingting Fan, Xiaojuan Zhang, Chen Feng, Yanbin Zhu, Yingze Zhang, Wei Chen

**Affiliations:** a Department of Orthopedic Surgery, Hebei Medical University Third Hospital, Shijiazhuang, Hebei, China; b School of Integrative Medicine, Tianjin University of Traditional Chinese Medicine, Tianjin, China; c Department of Nephrology, First Teaching Hospital of Tianjin University of Traditional Chinese Medicine, National Clinical Research Center for Chinese Medicine Acupuncture and Moxibustion, Tianjin, China

**Keywords:** O-GlcNAcylation, Heart, Kidney, Bones, Heart-kidney-bone axis, Inter-organ communication

## Abstract

The three major organ systems of heart, kidney and bone are highly correlated in physiology and pathology. They form the “heart-kidney-bone axis” together. The typical clinical manifestations of this axis include the deterioration of heart and kidney function, reflected by cardiorenal syndrome, renal bone disease, and cardiovascular calcification contained in chronic kidney disease-mineral bone disorder (CKD-MBD). However, the core molecular mechanism that regulates this axis has not been fully understood. O-linked N-acetylglucosamine (O-GlcNAc) glycosylation modification is a dynamic, reversible and highly conserved protein post-translational modification (PTM). It integrates body nutrition and stress signals via the hexosamine biosynthesis pathway (HBP), extensively participates in fine cellular function regulation, and plays an essential role in the progression of heart, kidney and bone diseases. This review systematically summarizes the role and multidimensional regulatory network characteristics of O-GlcNAcylation in heart, kidney and skeletal systems. It focuses on exploring its cross-organ regulatory mechanism in the “heart-kidney-bone axis”, and aims to provide new ideas for the mechanism analysis and targeted therapy of comorbidities.

## Highlight

• Proposing O-GlcNAc glycosylation modification as a molecular bridge integrating metabolic signals and inter organ regulation.

## 1 Preface

As highly prevalent and deadly diseases worldwide, heart [1], kidney [2] and bone diseases [3] seriously affect public health. Research has shown close pathophysiological connections between the three major systems of heart, kidney and bone [4]. Patients with chronic kidney disease (CKD) often develop cardiovascular disease and abnormal mineral bone metabolism [ 5,6]^.^ The occurrence and development of cardiovascular diseases are interrelated with bone metabolism imbalance and renal dysfunction [7,8]. Osteoporosis and its fracture events are significantly correlated with cardiovascular and renal function status as well [9,10]. This cross-system collaborative regulatory relationship has been summarized as the concept of the “heart-kidney-bone axis” [11]. Nevertheless, its underlying molecular mechanisms remain to be elucidated.

O-GlcNAcylation, an important intracellular modification widely expressed in heart, kidney and bone tissues, gets involved in regulating the functional homeostasis and disease progression of these organs [[12], [13], [14]]. It is jointly regulated by O-GlcNAc transferase and hydrolase (OGT and OGA, respectively). Its substrate UDP GlcNAc is derived from multiple metabolites (glucose, glutamine and acetyl CoA (Ac CoA)). Therefore, O-GlcNAcylation is also regarded as a “nutrient and stress receptor” of cells. It can dynamically respond to changes in energy status and affect transcriptional regulation, signal transduction, stress response and other key processes [[15], [16], [17]].

In the cardiovascular system, O-GlcNAc glycosylation exerts an influence on vascular calcification and myocardial function through the regulation of wingless-integrated/β(Wnt/β)-catenin and other signaling pathways, especially in diabetes cardiomyopathy [[18], [19], [20]]. In the kidney, this modification takes part in regulating podocyte function, fibrosis and inflammatory response, and affects the progression of acute and chronic kidney injuries [21]. In the skeletal system, O-GlcNAcylation participates in regulating bone remodeling and mineral metabolism by influencing the activity of osteoblasts and osteoclasts [22]. Its abnormality can also exacerbate the pathological effects of the heart-kidney-bone axis by mediating inter-organ signaling (e.g., bone-derived hormone disorders) [23,24].

**Fig. 1:**
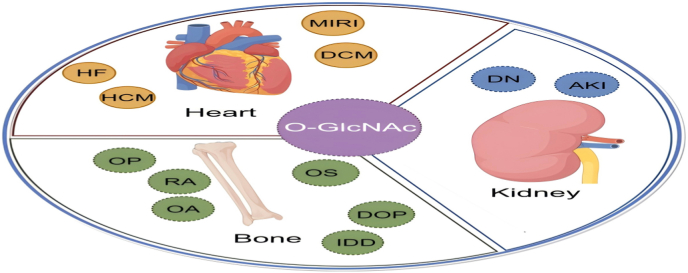
**Graphic and textual summary:**This figure illustrates the bridging role of O-GlcNAcylation in the “heart-kidney-bone axis.” O-GlcNAcylation integrates nutritional and stress signals via the hexosamine biosynthesis pathway (HBP) and dynamically regulates key cellular processes, including immune response, metabolic reprogramming, autophagy, and epigenetic regulation. Through these mechanisms, O-GlcNAcylation influences the physiological homeostasis of the heart, kidney, and bone, and mediates their pathological interactions under disease conditions such as cardiorenal syndrome, renal osteodystrophy, and cardiovascular calcification. This graphical summary highlights O-GlcNAcylation as a central cross-organ regulatory node and a potential therapeutic target for comorbidities involving the heart, kidney, and bone.

In summary, this review is aimed at systematically elucidating the bridging role of O-GlcNAcylation in the “heart-kidney-bone axis”(Fig. 1).Firstly, the basic characteristics and metabolic regulation mechanism of O-GlcNAcylation are introduced. Subsequently, a multidimensional regulatory network is constructed by targeting a variety of key intracellular processes to systematically influence organ homeostasis and disease development. Then, its physiological and pathological functions in heart, kidney and bone systems are summarized separately. Finally, the focus is on how O-GlcNAcylation mediates inter-organ interaction dialogue. By reviewing the research progress in this field, a new perspective is offered for understanding cross-organ regulatory networks, and new ideas are provided for preventing and treating related comorbidities.

## 2 Dynamic regulation of O-GlcNAcylation and the hexosamine biosynthesis pathway

OGT and OGA both maintain the dynamic balance of O-GlcNAcylation [25,26]. OGT is responsible for glycation addition, while OGA mediates glycation removal. The activity of OGT and OGA is regulated by nutritional status, post-translational modifications (PTMs) and protein-protein interactions [[27], [28], [29], [30]]. The availability of nutrients is the core factor that affects the glycosylation modification level of O-GlcNAc. The regulatory effect of O-GlcNAc is primarily achieved through the hexosamine biosynthesis pathway (HBP) [31].

**Fig. 2:**
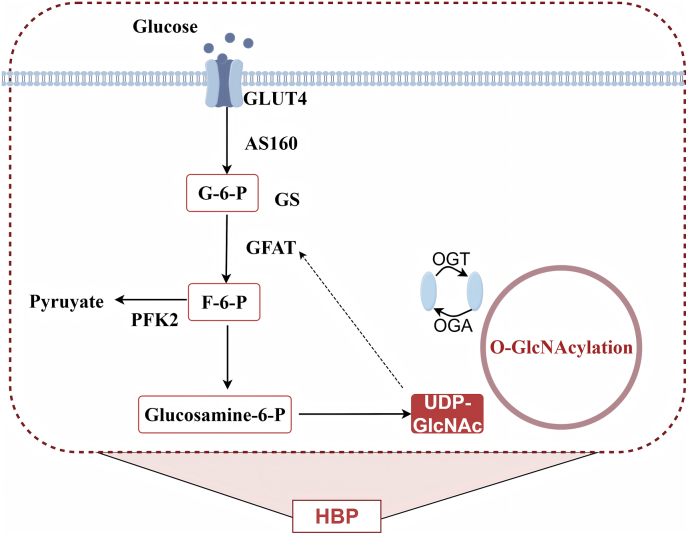
**Hexosamine biosynthetic pathway (HBP):**This diagram depicts the complete biosynthetic route from substrates (glucose, glutamine, acetyl-CoA, and UTP) to the final product UDP-GlcNAc, the essential donor substrate for O-GlcNAcylation. Key enzymes involved in each step are indicated, with glutamine-fructose-6-phosphate transaminase (GFAT) highlighted as the rate-limiting enzyme. Dashed arrows represent feedback inhibition, illustrating how the pathway maintains metabolic homeostasis. This figure provides a clear overview of HBP, which serves as the metabolic hub linking cellular nutrient status to protein O-GlcNAcylation.

HBP, as a branch pathway of glucose metabolism, is widely expressed in various tissues. It provides the essential substrate UDP GlcNAc for O-GlcNAcylation and serves as the core pathway for amino sugar biosynthesis [32]. Approximately 3% to 5% of glucose in the body enters HBP and participates in UDP GlcNAc synthesis together with glutamine, Ac CoA and uridine triphosphate (UTP) [33,34]. UDP GlcNAc, the final product of HBP, is a high-energy donor substrate for the biosynthesis of glycolipids, glycoproteins, proteoglycans and glycosaminoglycans [35]. HBP and glycolysis share the first two steps and enter different pathways at fructose-6-phosphate (F-6-P). The rate-limiting step is catalyzed by glutamine-fructose-6-phosphate transaminase (GFAT). F-6-P and glutamine are converted by GFAT into glucosamine-6-phosphate (GlcN-6-P) and glutamic acid [36]. This reaction is reversible and regulated by glucosamine 6-phosphate deaminase for the maintenance of the balance between GlcN-6-P and F-6-P [37]. Later, glucosamine phosphate N-acetyltransferase (GNPNAT) catalyzes the transfer of acetyl groups from Ac CoA to the primary amine of GlcN-6-P, which generates N-acetylglucosamine 6-phosphate (GlcNAc-6-P) [38,39]. GlcNAc-6-P is isomerized into N-acetylglucosamine 1-phosphate (GlcNAc-1-P) by means of phosphoglucose mutase 3 (PGM3/AGM1) [40]. Ultimately, UTP and GlcNAc-1-P in the nucleotide metabolism pathway are catalyzed by UDP-N-acetylglucosamine pyrophosphorylase 1 (UAP1/AGX1) for the generation of UDP-GlcNAc [[41], [42], [43]]. At the subcellular level, UDP GlcNAc is involved in N- and O-junction glycosylation in the Golgi apparatus and Golgi apparatus. OGT catalyzes protein O-GlcNAcylation in the cytoplasm and nucleus, and OGA takes charge of mediating modification removal [44,45]. In addition, free GlcNAc can be recovered back into HBP via the N-acetylglucosamine kinase mediated pathway, which maintains substrate homeostasis in the pathway [46](Fig. 2).

## 3 Multidimensional regulatory mechanism of O-GlcNAcylation

**Fig. 3:**
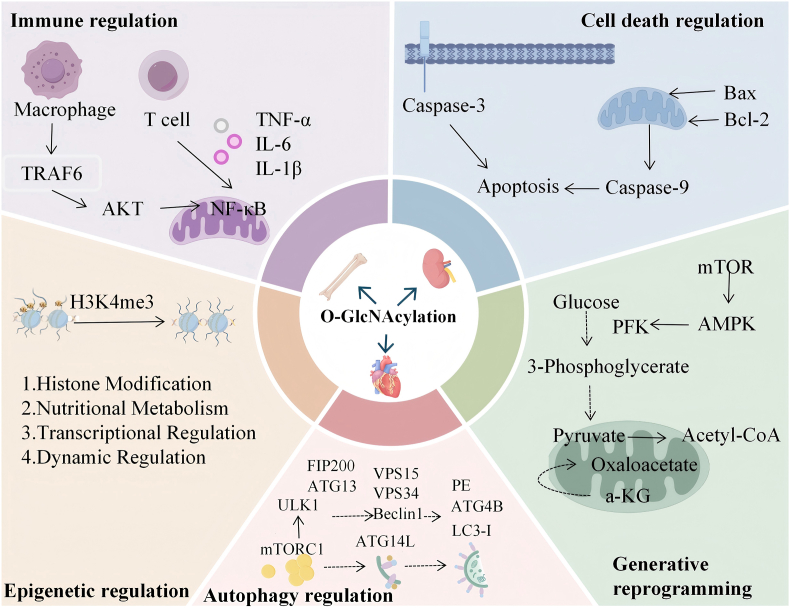
**Multidimensional regulatory mechanism of O-GlcNAcylation:**This figure summarizes the five major dimensions through which O-GlcNAcylation regulates cellular functions and disease progression: immune regulation, cell death control, metabolic reprogramming, autophagy regulation, and epigenetic regulation. By dynamically modifying key signaling molecules, transcription factors, and chromatin-associated proteins, O-GlcNAcylation integrates these processes into a multidimensional network that influences organ homeostasis in the heart, kidney, and bone. This regulatory network underlies both physiological maintenance and pathological deterioration, particularly in the context of inter-organ communication along the “heart-kidney-bone axis.”

O- GlcNAcylation plays an important role in maintaining physiological functions, pathological progression and the inter-organ interaction regulation of the “heart-kidney-bone axis”. Additionally, it constructs a multidimensional regulatory network by targeting multiple key intracellular processes, which thereby affects organ homeostasis and disease development in a systematic way(Fig. 3).

### 3.1 Immune regulation

Regulating the immune system precisely is the core guarantee for the body to resist pathogen invasion and avoid autoimmune damage. O-GlcNAc glycosylation is central in inflammation suppression and immune homeostasis maintenance through the dynamic modification of key signaling molecules in immune cells [[47], [48], [49]]. In macrophages, lipopolysaccharide (LPS) stimulation can induce the up-regulation of OGT expression. This in turn modifies the p65 subunit in the nuclear factor kappa-B (NF-κB) pathway to inhibit its nuclear translocation and transcriptional activation of downstream pro-inflammatory cytokines (e.g., tumor necrosis factor-alpha (TNF–α) and interleukin-6 (IL-6)). In this way, excessive inflammatory response is alleviated [[50], [51], [52]]. In the process of T cell activation, the O-GlcNAcylation levels of (NF-κB) and nuclear factor of activated T cells (NFAT) will correspondingly increase after the activation of T cell receptor (TCR). However, the activation process of T cells will be significantly inhibited after the elimination of OGT enzyme by small interfering ribonucleic acid (siRNA) [53]. Moreover, Jochmann et al. found that the high expression of OGT can raise the O-GlcNAcylation level of the specific protein 1 (Sp1) transcription factor in T cells, which thereby enhances the inhibition of human immunodeficiency virus (HIV) virus activity by T cells. This provides new evidence for the pathological significance of O-GlcNAcylation in regulating the function of T cells [54]. Furthermore, O-GlcNAc glycosylation modifies the protein glucose regulated protein 78 (GRP78) related to endoplasmic reticulum stress during the development of B cells and the secretion of antibodies. This reduces protein folding pressure during the activation of B cells and ensures the normal functioning of humoral immune function [21,55]. The above studies indicate that O-GlcNAcylation regulates the function of immune cells through multiple targets and pathways. Additionally, O-GlcNAcylation is a key molecular switch that connects inflammatory response and immune homeostasis.

### 3.2 Cell death regulation

Cell death is a critical biological process for maintaining tissue homeostasis and clearing abnormal cells, including apoptosis, necrotic apoptosis, etc. [56]. In apoptosis regulation, OGT can modify the serine residue of apoptosis executing protein Caspase-3 to suppress its enzymatic activity, which thereby blocks the initiation of the apoptosis cascade reaction [57]. Meanwhile, O-GlcNAcylation can also target B-cell lymphoma-2 (Bcl-2) family proteins. The modification of the anti-apoptotic protein Bcl-2 can enhance its stability, while the pro-apoptotic protein BCL-2-associated X protein (Bax) inhibits its mitochondrial localization and pore formation ability, which ultimately inhibits mitochondrial pathway apoptosis [58]. Unlike apoptosis regulation, O-GlcNAcylation in necrotic apoptosis is focused on the receptor-interacting serine/threonine-protein kinase 1 (RIPK1)-RIPK3-mixed-lineage kinase domain-like (MLKL) signaling axis [59]. OGT can modify the phospho-RIPK1 (Ser161) site of RIPK1 and inhibit its kinase activity and binding ability to RIPK3, which hence blocks the assembly of necrosomes [60,61]. In the meantime, O-GlcNAcylation can also down-regulate the phosphorylation level of MLKL, inhibit its membrane localization and perforation function, and finally block the execution of necrotic apoptosis [62,63].

### 3.3 Generative reprogramming

Metabolic reprogramming is a main feature of cellular adaptation to physiological needs (e.g., proliferation and differentiation) or pathological states (e.g., tumors and infections). O-GlcNAcylation serves as a “nutrient sensor” that is of importance to regulate the balance between glycolysis and the tricarboxylic acid (TCA) cycle by modifying metabolic enzymes and signaling molecules [64]. In the glycolysis pathway, O-GlcNAc glycosylation can target key enzyme hexokinase 2 (HK2) [65], phosphofructokinase 1 (PFK1) 66 and pyruvate kinase M2 (PKM2). The modification of HK2 can enhance its mitochondrial binding ability and promote glucose phosphorylation [65]. The modification of PFK1 restrains its activity and reduces the diversion of glycolytic intermediates to the TCA cycle [66]. The modification of PKM2 can induce its nuclear translocation and act as a transcriptional co-activator to regulate the expression of glycolysis-related genes. This ultimately promotes glycolysis enhancement (i.e., the “Warburg effect”) and meets the energy requirements of rapidly proliferating cells like tumor cells. In the regulation of the TCA cycle, O-GlcNAc glycosylation mainly works by modifying isocitrate dehydrogenase (IDH) [67] and α-ketoglutarate dehydrogenase complex (α-KGDHC) [68]. OGT can modify the Ser113 site of IDH1, which inhibits its ability to catalyze the generation of α-ketoglutarate and reduces the flux of the TCA cycle. At the same time, O-GlcNAcylation can enhance the stability of α-KGDHC, promote the production of succinic acid and maintain the function of the mitochondrial respiratory chain [68]. More than that, O-GlcNAc glycosylation can indirectly regulate the balance between glycolysis and the TCA cycle by modifying metabolic signaling pathway molecules like adenosine 5′-monophosphate-activated protein kinase (AMPK) and mammalian target of rapamycin (mTOR) [69,70]. The activation of AMPK can promote OGT activity and enhance glycolysis. On the other hand, mTOR reduces the consumption of TCA cycle intermediates by inhibiting the expression of OGT [69,70]. These studies demonstrate that O-GlcNAcylation becomes a core regulatory factor connecting cellular nutritional status and metabolic reprogramming through “directly modifying metabolic enzymes and indirectly regulating signaling pathways” [[71], [72], [73]].

### 3.4 Autophagy regulation

Autophagy is a crucial process where cells clear damaged organelles and misfolded proteins under stress conditions like oxidative stress and nutrient deficiency [74]. For the past few years, research has found the unique regulatory role of O-GlcNAcylation in activating oxidative stress-induced autophagy, and provided a new perspective for studying autophagy mechanisms [75,76]. Under oxidative stress conditions, an increase in intracellular reactive oxygen species (ROS) levels can induce the up-regulation of OGT expression and O-GlcNAc levels [77], which thereby exerts regulatory effects by modifying key proteins in autophagy initiation complexes. OGT can modify the Ser757 site of Unc-51-like autophagy activating kinase 1 (ULK1), inhibit the mTOR phosphorylation inhibition of ULK1, and facilitate ULK1 activation [78,79]. O-GlcNAc glycosylation can modify the Ser90 site of Beclin1, enhance its binding ability to vacuolar protein sorting 34 (Vps34), promote phosphatidylinositol 3-kinase (PI3K)-III complex assembly and initiate autophagosome formation [80,81]. During autophagosome maturation, O-GlcNAc glycosylation can modify the Ser12 site of microtubule-associated protein light chain 3 (LC3) and enhance its interaction with autophagy receptor p62. Moreover, it can promote the encapsulation and degradation of damaged targets like mitochondria (mitochondrial autophagy) and the endoplasmic reticulum (endoplasmic reticulum autophagy) [82,83]. O-GlcNAcylation may be a “protective regulatory factor” maintaining cellular homeostasis under oxidative stress conditions, which provides a new target for autophagy-targeted intervention in related diseases [84,85].

### 3.5 Epigenetic regulation

O-GlcNAcylation, an integral component of epigenetic regulatory networks, is involved in gene expression regulation through multidimensional mechanisms. It can directly modify histones and transcription factors at the level of histone modification interaction, which contributes to the formation of a key link in the epigenetic regulatory network [86]. Previous research has confirmed that the O-GlcNAc modification of chromatin factors plays a vital role in the transcriptional inhibition of methylated retrotransposons. Even in the absence of deoxyribonucleic acid (DNA) methylation loss, local chromatin deglycosylation can specifically activate the expression of targeted retrotransposon families [87]. Simultaneously, this modification can also epigenetically regulate gene expression like the “histone code” by regulating histone markers like trimethylated histone H3 at lysine 27 (H3K27me3) [88]. As a PTM sensitive to nutritional status, O-GlcNAc glycosylation further builds a regulatory bridge between nutritional metabolism and epigenetic reprogramming [89]. Among them, OGT is the only enzyme capable of adding GlcNAc groups to serine/threonine hydroxyl sites. Whole genome analysis shows that its mediated glycosylation protein can bind to active retrotransposon promoters and imprinting control regions together with factors like tripartite motif-containing 28 (TRIM28) [87]. During development, this modification exhibits higher responsiveness to nutrient disturbance signals and regulates epigenetic dynamics in a direct way [90]. At the level of transcriptional regulation, the mechanism of O-GlcNAc modification is multifaceted: O-GlcNAc modification can not only directly modify RNA polymerase II and transcription factors [91,92]. Moreover, the divalent modification of histones in the promoter region can be disturbed (e.g., increasing histone H3 (1-20), trimethylated lysine 43 (H3K4me3) levels) by regulating the phosphorylation status of enhancer of zeste homolog 2-estrogen receptor alpha (EZH2-Thr311) [90]. In addition, O-GlcNAc modification can synergistically mediate epigenetic silencing with polycombin complex (PcG), which is crucial for the developmental regulation and maintenance of stem cell homeostasis [93]. Besides, it can also synergistically form a dual epigenetic modification network of DNA and histones with Tet methylcytosine dioxygenase 2 (TET2) [94]. From the perspective of dynamic regulation characteristics, O-GlcNAc glycosylation and phosphorylation exhibit a “yin-yang regulation” relationship on the same protein [95]. Its modification cycle is strictly regulated by OGT and OGA (glycosylation and deglycosylation enzymes, respectively) [96]. This dynamic modification can turn into a core hub linking cellular metabolic status to epigenetic regulation by affecting transcriptional complex assembly, chromatin-modifying enzyme activity, and other pathways [97,98]. To sum up, O-GlcNAc glycosylation is not only a passive target for epigenetic regulation, but also a key modification system for actively shaping the cellular epigenetic landscape [99].

## **4** **Role of O-glycosylation modifications in the “heart**-**kidney**-**bone axis”**

### 4.1 O-GlcNAcylation in the heart

#### 4.1.1 Maintenance of physiological functions

Under physiological conditions, the O-GlcNAcylation of the heart maintains a moderate level and regulates the function and signaling pathways of key cardiac proteins for the purpose of maintaining cardiac systolic and diastolic activity and electrophysiological stability [100]. O-glycosylation modification can regulate contraction-related proteins like troponins T (TnT and TnI), and maintain their interaction efficiency with actin and myosin by changing their spatial conformation. Beyond that, it can ensure force transmission in myocardial excitation-contraction coupling, and stabilize cardiac pumping function [101,102]. O-GlcNAcylation also has the capacity to strengthen the structural stability of TnT, reduce degradation and avoid the weakening of myocardial contractility caused by insufficient contractile proteins [101]. At the level of signaling pathways, O-GlcNAcylation and phosphorylation regulate the activity of calmodulin dependent kinase II (CaMKII) synergistically, and maintain its physiological range to maintain calcium homeostasis and normal heart rhythm [103,104]. More than that, this modification promotes the expression of cardiomyocyte-specific genes by regulating the transcription factor GATA binding protein 4 (GATA4). GATA4 has important effects on the survival, functional maintenance and cardiac development of cardiomyocytes [105].

#### 4.1.2 Pathological effects

The balance of O-GlcNAc modification is usually destroyed in the pathological process of heart failure (HF), myocardial hypertrophy, myocardial ischemia/reperfusion injury and diabetes cardiomyopathy. In addition, the level of O-GlcNAc modification is abnormally elevated or reduced, and actively participates in disease progression [106,107].

In HF, abnormal O-GlcNAcylation levels are closely associated with worsening heart function. O-GlcNAcylation in the myocardium of OGT transgenic mice is greatly elevated, which makes them prone to developing dilated cardiomyopathy (DCM) and ventricular arrhythmia and suffering from sudden death [108]. On the contrary, OGA transgenic mice showed reduced modification levels and resistance to stress-induced myocardial injury, with milder pathological hypertrophy [109]. Mechanistically, excessively modifying O-GlcNAc leads to mitochondrial dysfunction and energy metabolism disorders, while partially reducing modification levels can improve the activity of mitochondrial complex I and alleviate myocardial lesions [110]. In addition, OGT knockout, giving rise to O-GlcNAc deficiency in myocardial cells, inhibits autophagy and reduces the modification level of autophagy initiating kinase ULK1, which indicates the key role of this modification in regulating myocardial autophagy [111].

During the course of myocardial hypertrophy, the abnormal activation of the HBP pathway and the subsequent increase in O-GlcNAcylation are pivotal promoting factors [112,113]. Pathological stimulation up-regulates the activity of rate-limiting enzymes like GFAT1, increases the generation of UDP-GlcNAc, and thus enhances the level of modification [113,114]. High-level O-GlcNAcylation activates the expression of hypertrophy-related genes like atrial natriuretic peptide (ANP) and brain natriuretic peptide (BNP) by promoting the nuclear translocation of transcription factors like NFAT and GATA4 [112,115,116]. Meanwhile, the modification of troponin I (TnI) can alter its phosphorylation status, reduce calcium sensitivity, disrupt contraction coordination and accelerate pathological remodeling [112,116].

O-GlcNAcylation plays a context-dependent role in myocardial ischemia/reperfusion injury [117,118]. During the phase of ischemic preconditioning, the moderate elevation of modifications can activate antioxidant enzymes (e.g., superoxide dismutase (SOD)) and stabilize heat shock proteins (e.g., heat shock protein 70 (Hsp70)), alleviate oxidative stress and endoplasmic reticulum stress. Hence, this enhances cellular tolerance [[118], [119], [120], [121]]. In severe or late reperfusion, excessive modification can result in the dysfunction of calcium processing proteins, the abnormal activation of apoptotic pathways and the massive accumulation of ROS, which further disrupts the OGT/OGA balance and exacerbates myocardial injury [117,[122], [123], [124]].

Diabetes cardiomyopathy is closely related to abnormal O-GlcNAc glycosylation resulting from long-term hyperglycemia [125,126]. High blood sugar promotes glucose influx into the HBP pathway, which increases UDP GlcNAc production and modification levels [127,128]. Excessive modification continuously activates CaMKII, which causes calcium overload and cell apoptosis [19,129]. Inhibiting the function of the mitochondrial respiratory chain leads to reduced adenosine triphosphate (ATP) synthesis and ROS accumulation [130,131]. The modification of insulin receptor substrate-1 (IRS-1) impairs insulin signaling transduction, which exacerbates myocardial metabolic disorders [105,132].

### 4.2 O-GlcNAcylation in kidneys

#### 4.2.1 Maintenance of physiological functions

Kidneys play a central role in the clearance of metabolic waste and the homeostasis of the internal environment. O-GlcNAcylation exerts a huge impact on this function by regulating cellular signaling and substance transport processes [12,133]. In the glomerulus, this modification regulates capillary hemodynamics and cell contraction by acting on the cytoskeleton proteins of mesangial cells, which thereby maintains normal filtration function [12,133]. In podocytes, O-GlcNAcylation is beneficial to stabilizing podocyte-associated proteins and filter barrier integrity, which prevents protein leakage [134]. In renal tubules, sodium glucose cotransporter 2 (SGLT2) in proximal tubular epithelial cells is regulated by O-GlcNAcylation. This maintains the membrane expression and activity of SGLT2 within physiological limits, which ensures glucose reabsorption and glucose metabolism balance [135]. O-GlcNAcylation also participates in regulating renal stress and reduces oxidative damage by enhancing the activity of antioxidant enzymes and regulating inflammatory signaling molecules (e.g., NF-κB), inhibiting excessive inflammation and maintaining renal homeostasis [12,133,136].

#### 4.2.2 Pathological effects

Renal diseases like diabetic nephropathy and acute renal injury are generally accompanied by abnormal O-GlcNAcylation that gets involved in the disease process by affecting filtration, transport and inflammatory response [133,137].

Diabetic nephropathy, the major microvascular complication of diabetes, is characterized by glomerulosclerosis, proteinuria and podocyte damage. Its pathogenesis is closely correlated with the increase of O-GlcNAc glycosylation levels [137,138]. Long-term hyperglycemia leads to the excessive activation of the HBP pathway, the up-regulation of GFAT1 expression, the increased production of UDP GlcNAc, and the abnormal elevation of modification levels [137,139]. In mesangial cells, excessive modification activates the transforming growth factor-β(TGF-) signaling pathway: the modification of TGF-βreceptor enhances activity, promotes the nuclear translocation of mothers against decapentaplegic homolog 2/3 (Smad2/3), up-regulates the expression of extracellular matrix proteins like collagen IV and fibronectin, and expedites mesangial dilation and glomerulosclerosis [137,139]. In podocytes, abnormal modifications disrupt the function of the perin policin complex, undermine the integrity of the diaphragm, induce proteinuria and promote cell apoptosis, which exacerbates glomerular dysfunction [137,140]. Additionally, O-GlcNAcylation promotes the nuclear translocation of NF-κB p65, up-regulates the expression of inflammatory factors like TNF-αand IL-6, which further aggravates kidney inflammation and injury [141,142].

Proximal tubule injury is a central link in the pathophysiology of diabetic kidney disease (DKD), with tubular atrophy and interstitial fibrosis closely associated with impaired renal function and poor prognosis in patients [143]. Prolonged hyperglycemia leads to increased glucose uptake by glomerular and proximal tubule cells, enhancing the flux of the hexosamine biosynthesis pathway (HBP). Its end product, UDP-GlcNAc, drives rapid and reversible O-GlcNAc modification (O-GlcNAcylation) of thousands of intracellular proteins. This modification is significantly elevated in both diabetic and non-diabetic chronic kidney disease. Damaged proximal tubule cells mediate monocyte/macrophage recruitment via O-GlcNAcylation-induced inflammatory signaling, exacerbating local inflammatory responses [144]. Additionally, the activation of the TGF-β1/Smad3 and NF-κB pathways jointly contributes to renal fibrosis progression, while inhibiting terminal fucosylation can alleviate renal fibrosis [145,146]. Functionally, O-GlcNAcylation can directly affect the activity of key proteins: for instance, abnormal glycosylation of megalin impairs its endocytosis and reabsorption functions, thereby disrupting the reabsorption of substances such as albumin [147].

As a common, heterogeneous and multifactorial disease, acute kidney injury (AKI) is a part of acute kidney disease and syndrome disorders [148]. O-GlcNAcylations exhibit dynamic changes during the occurrence and development of AKI [149]. The activity of autophagy-related proteins (e.g., LC3) is enhanced to promote the clearance of damaged components. Molecular chaperone proteins (e.g., Hsp90) are modified to improve cell stress resistance and alleviate oxidative damage and apoptosis [82]. During ischemia/reperfusion injury, a large amount of ROS is produced to disrupt OGT/OGA activity. This leads to fluctuating modification levels and further affects cellular function and repair processes, which ultimately limit the prognosis of AKI [21,[149], [150], [151]].

### 4.3 O-GlcNAcylation in bones

#### 4.3.1 Maintenance of physiological functions

Bones not merely provide mechanical support, but also participate in mineral metabolism and hematopoietic regulation [152]. Their homeostasis is up to the dynamic balance between osteoblast-mediated bone formation and osteoclast-mediated bone resorption [153], where O-GlcNAcylation is important [154,155].

In osteoblasts, O-GlcNAcylation promotes osteogenic differentiation through regulating the activity of key transcription factors like Runt-related transcription factor 2 (Runx2) [156] and Osterix [157,158]. After modification, it can enhance its binding ability with bone matrix protein gene promoters like osteocalcin and type I collagen, which thereby facilitates the synthesis and secretion of bone matrices [159]. Moreover, this modification is also conducive to maintaining the mitochondrial function of osteoblasts and provides necessary energy support for bone matrix mineralization [160,161].

In osteoclasts, O-GlcNAcylation prevents excessive bone loss via the fine regulation of their differentiation and bone resorption activity [162]. Moderate modification can inhibit signaling pathways related to the abnormal differentiation of osteoclasts, which thereby limits their excessive activation [163,164].

In bone marrow stromal cells (BMSCs), O-GlcNAcylation promotes their differentiation into osteoblasts by activating signaling pathways like Wnt/β-catenin, while suppressing their differentiation into adipocytes. This helps maintain the stability of the osteoblast pool and guarantees the normal process of bone remodeling [22,158,165].

#### 4.3.2 Pathological effects

Under pathological conditions such as aging, hyperglycemia and hormone disorder, the imbalance of O-GlcNAc glycosylation will destroy the balance of bone remodeling. It will also participate in the occurrence and development of osteoporosis [165,166], osteoarthritis [165,166] and osteosarcoma [166], and the degeneration of intervertebral disc, diabetes osteopathy [167], etc.

Osteoporosis features reduced bone mass, increased bone fragility and destroyed bone microstructure. Its core pathology is bone resorption exceeding bone formation [168]. The abnormal glycosylation modification of O-GlcNAc (manifesting as an increase or decrease, depending on the disease stage and trigger) exacerbates the condition by influencing the function of osteoblasts and osteoclasts [164,169]. In terms of osteogenesis, excessive modification can have an inhibiting effect on the transcriptional activity of Runx2 and Osterix, which reduces bone matrix synthesis [170,171]. Inadequate modification can damage the Wnt/β-catenin signaling pathway and hinder osteogenic differentiation. Both of them lead to impaired osteoblast function and mineralization disorders [172]. Regarding bone resorption, excessive modification can enhance NF-κB and mitogen-activated protein kinase (MAPK) signaling, promote the differentiation and activation of osteoclasts, prolong their survival time, and thus exacerbate bone resorption [163,173].

Osteoarthritis (OA) and rheumatoid arthritis (RA) are also linked to abnormal O-GlcNAcylations [174]. Elevated levels of modification in OA cartilage can induce pathological chondrocyte hypertrophy and promote synovial inflammation through the TGF-β pathway [175]. In RA, the O-GlcNAcylation of p65 subunit enhances the inflammatory response induced by TNF-α in synovial cells [162] As a result, O-GlcNAcylation has become a potential therapeutic target for OA and RA.

In osteosarcoma, O-GlcNAcylation exerts an impact on tumor progression and chemotherapy response via the regulation of gene expression and signaling pathways [176]. The high expression of OGT is associated with non-metastatic and chemotherapy resistance [177].Mechanistically, this modification activates the Rho-associated coiled-coil containing protein kinase 2 (ROCK2) pathway to regulate apoptosis, which thereby promotes a drug resistance phenotype [178].

In intervertebral disc degeneration (IDD), O-GlcNAcylation and OGT/OGA expression levels are both elevated [179]. Glucose invasion through endplate vessels can activate HBP, enhance sex-determining region Y box 9 (Sox9) and Runx2 modifications: inhibit the function of Sox9 to hinder cartilage differentiation, enhance the function of Runx2 to promote ectopic osteogenesis, and jointly accelerate degeneration [180].

Diabetes osteopathy is hallmarked by decreased bone density, damaged bone quality and increased fracture risk, which is closely bound up with abnormal O-GlcNAc glycosylation attributed to long-term hyperglycemia [181]. Hyperglycemia activates HBP, which leads to over-modification. It causes damage to bones through the following mechanisms [181], reduces the transcriptional activity of Runx2 and Osterix [170,171], and speeds up the process of diabetes osteopathy.

### 4.4 Cross-organ regulatory mechanisms in the “heart-kidney-bone axis”

The “heart-kidney-bone axis” refers to the functional regulatory network established by the mutual influence between the heart, kidneys and bones by means of neural, humoral and metabolic pathways. The three jointly maintain the homeostasis of the internal environment of the body. From the angle of Western medicine, this axis relies on the hypothalamic-pituitary-ovarian axis (HPOA) [[182], [183], [184], [185]] and the renin-angiotensin aldosterone system (RAAS) [186,187] to form a neural endocrine metabolic regulatory network and achieve cross-organ signal transmission. As a key sensor for cellular nutrition and metabolic status, O-GlcNAcylation not only regulates physiological and pathological processes within various organs, but also participates in overall homeostasis regulation through cross-organ signal transmission via the axis system. The imbalance of O-GlcNAcylations can also exacerbate multi-organ diseases through cross-organ effects [71,188,189], which forms a vicious cycle of “the imbalance of organ damage modifications leads to multi-organ involvement”.

Cardiorenal syndrome (CRS) is defined as a bidirectional disorder in which primary dysfunction of either the heart or the kidney induces secondary injury to the other [190].The core mechanisms include sustained activation of the renin-angiotensin-aldosterone system (RAAS), chronic inflammation, oxidative stress, mitochondrial dysfunction, and tissue fibrosis and remodeling.In the early repair phase, cellular proliferation and polyploidy occur, but these eventually progress to pathological fibrosis, further worsening both cardiac and renal function [191].Although a five-type classification based on the temporal sequence of cardio-renal events is used clinically, its methodological and practical utility remains debated [192].Notably, CRS is only one component of the broader field of “nephrocardiology”, which encompasses nine dimensions including pathophysiology, epidemiology, prevention, prognosis, and diagnosis [193].Epidemiologically, the coexistence of heart failure and chronic kidney disease (CKD) dramatically increases hospitalization rates, treatment complexity, and adverse outcomes, representing a major challenge in the management of cardio-renal comorbidities [7].

Renal osteodystrophy (ROD) is the skeletal manifestation of chronic kidney disease-mineral and bone disorder (CKD-MBD), characterized by abnormal bone mineralization and remodeling [194].Its pathogenesis involves disordered mineral homeostasis (e.g., hyperphosphatemia, secondary hyperparathyroidism) and accumulation of uremic toxins.Uremia itself impairs osteocyte viability and induces skeletal resistance to parathyroid hormone (PTH).PTH exerts compartment-specific effects: it protects trabecular bone but is detrimental to cortical bone [195].Emerging evidence suggests that stalled mitophagy in osteocytes may contribute to bone loss [196].The main clinical phenotypes are high-turnover disease (driven by secondary hyperparathyroidism) and low-turnover (adynamic) bone disease, with the latter now being the most common type and strongly associated with poor prognosis [197].Traditional anti-resorptive agents (e.g., bisphosphonates) require caution or avoidance due to impaired renal clearance, whereas calcimimetics (e.g., etelcalcetide) target the PTH pathway for high-turnover ROD [198,199].Epidemiologically, in the German CKD (GCKD) study (n = 5217), multiple MBD biomarkers (e.g., PTH, phosphate) were significantly associated with cardiovascular events and mortality [200].ROD markedly increases fracture risk and all-cause mortality in CKD patients, contributing substantially to morbidity and reduced quality of life [194].

Cardiovascular calcification is a key component of CKD-MBD, driven by mineral imbalance (particularly hyperphosphatemia), chronic inflammation, uremic toxins, and disturbed bone metabolism [201].These factors induce vascular smooth muscle cell (VSMC) transdifferentiation into an osteoblast-like phenotype, promoting hydroxyapatite deposition, arterial stiffness, and cardiovascular events [202].Abnormal serum phosphate is an independent risk factor for adverse cardiovascular outcomes [203].Interventions that lower bone turnover (e.g., calcimimetics, parathyroidectomy) may influence vascular calcification progression, underscoring the tight bone-vascular axis [197].Epidemiologically, patients with CKD-MBD have an “extremely high” risk of cardiovascular disease, especially ischemic heart disease.The presence of vascular calcification directly correlates with increased mortality risk.CKD itself is considered a “high or very high cardiovascular risk state”, and CKD-MBD is a core driver of this risk [204].Together, the burden of fractures, reduced quality of life, and cardiovascular disease account for the high morbidity and mortality in CKD patients [201].

#### 4.4.1 Heart-kidney interaction

The heart and kidneys form “cardiorenal syndrome” through hemodynamic and neuroendocrine pathways. Dysfunction in one normally results in secondary damage in the other [205]. O-GlcNAcylation plays a critical regulatory role in this interaction.

In cardiogenic kidney injury, decreased cardiac pumping function leads to inadequate renal perfusion, which activates RAAS. The activation of RAAS further promotes the activation of HBP in renal cells, enhances OGT activity, inhibits OGA activity, and increases O-GlcNAcylation levels [144,206]. High levels of modification intensify kidney damage through a dual mechanism, enhance the activity of kidney transporters like sodium-dependent glucose transporters 2 (SGLT2) and sodium-hydrogen exchanger 3 (NHE3), increase water and sodium reabsorption and retention, and further elevate heart load [125,207]. The modification of proteins related to inflammatory fibrosis, like TGF-βand NF-κB, promotes renal inflammation and fibrosis, which exacerbate renal dysfunction [133,208]. Renal dysfunction, in turn, increases blood volume, raises blood pressure and produces uremic toxins. This exacerbates the burden on the heart [209], which forms a vicious cycle of mutual damage to the heart and kidneys.

In CKD-induced cardiac injury, renal dysfunction triggers pathological changes like oxidative stress. These pathological changes activate HBP after entrance into myocardial cells, which leads to an increase in O-GlcNAcylation levels [210,211]. Over-modification promotes cardiac injury through multiple pathways. It promotes an increase in myocardial cell volume by modifying transcription factors related to myocardial hypertrophy, like GATA4 and NFAT [212]. Myocardial interstitial fibrosis is increased by modifying proteins related to collagen synthesis [213]. Cardiac calcium homeostasis is disrupted and arrhythmia is induced by modifying proteins related to calcium processing, like sarcoplasmic reticulum calcium ATPase 2A (SERCA2a) and CaMKII [106].

#### 4.4.2 Renal-bone interaction

Kidneys maintain bone homeostasis through the regulation of calcium and phosphorus metabolism and the activation of vitamin D. Bones affect kidney function through bone-derived hormones, which leads to the formation of the “renal-bone axis” [214]. The imbalance of O-GlcNAcylation is a crucial trigger for renal bone disease.

In the process of CKD causing renal bone disease, renal dysfunction results in calcium and phosphorus metabolism disorders, vitamin D activation disorders as well as secondary hyperparathyroidism. These pathological changes affect the O-GlcNAcylation status of kidneys and skeletal cells. At the renal level, decreased renal function inhibits OGA activity, which increases O-GlcNAcylation levels [215]. Additionally, it modifies calcium phosphate transporters (e.g., calcium binding proteins and sodium phosphate co-transporters) in renal tubular epithelial cells, restrict their activity, reduce calcium reabsorption and phosphorus excretion, and aggravate hyperphosphatemia and hypocalcemia [216]. At the skeletal level, the HBP of bone cells can be activated by hyperphosphatemia, hypocalcemia and the deficiency of active vitamin D, which leads to the abnormal elevation of O-GlcNAcylation. This modifies osteoblasts Runx2 and Osterix, inhibit their transcriptional activity, and reduce bone matrix synthesis [170,171]. Osteoclasts are modified with NF-κB and MAPK to enhance their activity, which promotes osteoclast differentiation and bone resorption, and leads to decreased bone mass and structural damage [163,173]. Thus, a metabolically driven kidney-bone loop is established: O-GlcNAcylation links disordered mineral metabolism to abnormal bone remodeling, while bone-derived cytokines (e.g., TGF-β, IL-6) further elevate O-GlcNAcylation in renal tubular cells, aggravating the same mineral disturbances and perpetuating bidirectional injury.

#### 4.4.3 Heart-bone interaction

The heart and bones form a “cardioskeletal axis” through hormone secretion and metabolic signals. Cardiac natriuretic peptides have an influence on bone metabolism, while bone calcium regulates heart function [217]. O-GlcNAcylation is involved in axis signaling, and its imbalance intensifies heart and bone diseases through cross-organ effects.

When HF causes bone damage, the pumping function of the heart decreases, which leads to the insufficient perfusion of bone tissue. HF-related malnutrition and oxidative stress affect the metabolic status of bone cells simultaneously. These pathological factors activate HBP in skeletal cells, which increases O-GlcNAcylation levels. Osteoblasts Runx2 and Osterix are modified to inhibit osteogenic differentiation and bone matrix synthesis [170,171]. The modification of osteoclasts with NF-κB and MAPK promotes osteoclast activation and bone resorption, which results in bone loss and osteoporosis [163,173].Thus, a heart-bone endocrine-metabolic axis is established: heart failure-induced O-GlcNAcylation in osteoblasts suppresses osteocalcin secretion, and the subsequent decline in circulating osteocalcin impairs myocardial insulin sensitivity and mitochondrial oxidative metabolism, thereby amplifying bidirectional cardiac and skeletal damage. In this axis, O-GlcNAcylation-mediated osteocalcin reduction serves as the key molecular link between cardiac dysfunction and bone loss.

When osteoporosis causes heart damage, the secretion of osteocalcin is reduced in patients with osteoporosis. Osteocalcin, a key bone-derived hormone, has the effect of regulating energy metabolism and improving insulin sensitivity [218]. The reduction of osteocalcin causes systemic metabolic disorders, which in turn affect the metabolism of myocardial cells [219]. The insulin signaling pathway of myocardial cells is inhibited; HBP is activated; an increase occurs in the level of O-GlcNAcylation [220]. Excessive modification aggravates myocardial metabolic disorders, promotes myocardial hypertrophy and fibrosis, and deteriorates cardiac function [127].

#### 4.4.4 Heart-kidney-bone interaction

Damage to any organ of the heart, kidneys or bones can have a bearing on the other two through abnormal modifications. The synergistic damage of the three further amplifies the imbalance of modifications. Classic examples include “CKD combined with heart and bone lesions” and “perimenopausal osteoporosis (PMOP) combined with heart and kidney dysfunction”.

When PMOP is accompanied by cardiac and renal dysfunction, a decrease in estrogen levels can directly activate the HBP pathway in bone cells, which leads to an increase in O-GlcNAc modification, inhibits osteoblast activity and promotes the activation of osteoclasts [22]. The decrease of osteocalcin further affects myocardial energy metabolism, activates myocardial HBP, enhances O-GlcNAc modification and triggers myocardial hypertrophy and fibrosis [23,221]. Reduced myocardial pumping function leads to renal hypoperfusion, which activates RAAS and further renal HBP, exacerbates O-GlcNAc modification abnormalities, and causes inflammatory fibrosis and renal calcium phosphate transport disorders [18,222]. The deterioration of renal function is mediated by disturbances in calcium and phosphorus metabolism. In addition, the impaired activation of vitamin D enhances skeletal O-GlcNAc modification and forms a cycle of “estrogen deficiency → multiple organ dysregulation → synergistic damage” [22,155].

CKD in CKD combined with cardiac and skeletal lesions leads to disturbances in calcium and phosphorus metabolism and impaired activation of vitamin D, which directly activates the HBP pathway in kidneys and bones and enhances O-GlcNAc modification [18,222]. Abnormal bone modification further decreases osteocalcin secretion and affects myocardial energy metabolism [22,223]. The reduction of osteocalcin inhibits myocardial energy metabolism, activates myocardial HBP, leads to increased O-GlcNAc modification, facilitates myocardial remodeling and decreases cardiac function [23,224]. The deterioration of heart function exacerbates renal hypoperfusion, which further damages renal function 104, 204. This process forms a feedback loop of “renal injury → abnormal cardiac bone modification → further deterioration of renal function” [18,222].

## 5 Summary and prospect

As a cellular nutrient and stress receptor, O-GlcNAcylation is dynamically balanced by OGT/OGA ^25^and integrates metabolic signals such as glucose, glutamine and Ac CoA through HBP [[32], [33], [34]]. It becomes a key molecular node that connects cellular metabolic status and organ functional homeostasis [12]. O-GlcNAcylation plays an irreplaceable role in the physiological regulation and pathological progression of the heart-kidney-bone axis [22,106,107,133,155]. This function is orchestrated through a multidimensional regulatory network that includes immune modulation [[47], [48], [49]], metabolic reprogramming [[64], [65], [66]], autophagy regulation [75,83], and epigenetic control [87,89], enabling the integration of cellular nutrient and stress signals with organ-specific homeostasis [15,16].

The balance of cardiac contractile function, renal filtration homeostasis and bone remodeling can be maintained and become a key molecular bridge connecting cardiorenal syndrome, renal osteopathy, diabetes osteopathy and other comorbidities in the following ways: modifying cardiac contractile proteins (TnT and TnI) [127,208], kidney-related proteins (Nephrin-Podocin complex) [216] and bone differentiation transcription factors (Runx2 and Osterix) [155]. This review systematically summarizes the regulatory network of this modification in the axis system, which provides a theoretical framework for studying cross-organ pathological mechanisms. Further exploration and refinement are needed in the future.

However, it remains unclear about the specificity and mechanism details of O-GlcNAc modification in axis regulation. In the heart-kidney interaction, the modification sites of RAAS pathway-related proteins renin or angiotensin II receptors may be different, but the direct evidence of “site function” is lacking at present [225]. It is still necessary to verify whether the O-GlcNAc modification of proteins related to calcium phosphate metabolism (e.g., Calbindin-D28k and PiT1) affects cross-organ signaling through different mechanisms in kidney bone interaction [71]. This limits the precise differentiation of organ-specific effects of modified proteins to a large extent. The synergistic effect of multidimensional regulatory mechanisms has not been expounded. The synergistic or antagonistic relationship between immune regulation mediated by O-GlcNAc modification, metabolic reprogramming and autophagy regulation in comorbidities like perimenopausal osteoporosis with cardiac and renal dysfunction is unclear. The overall regulation of the disease is not understood, which restricts the development of multi-target intervention strategies.

Future research can promote the translation from basic to clinical practice: focusing on organ-specific delivery of OGT/OGA targeted drugs is necessary at the level of precision therapy. The off-target effects of systemic administration are reduced through the delivery of OGT inhibitors (e.g., OGT inhibitor (OSMI-1)) through cluster of differentiation (CD47) receptors highly expressed in cardiomyocytes [226] or the targeted delivery of OGA inhibitors (e.g., Thiamet-G) through osteogenic bone morphogenetic protein receptors (BMPRs) [160,227]. In terms of biomarker development, O-GlcNAc-modified protein is expected to become a diagnostic tool for comorbidities. In cardiorenal syndrome, modified TnI (a marker of myocardial injury) and modified nephrin (a marker of glomerular injury) may serve as combined diagnostic indicators [228]. In CKD-MBD, the ratio of modified Runx2 (an index of osteogenic function) to modified Cathepsin K (an index of osteoclast activity) can reflect the imbalance of bone remodeling more accurately [155].

To conclude, O-GlcNAc glycosylation has become a core regulatory node for multi-system comorbidities by integrating metabolic signals and cross-organ dialogue [187,227]. Future studies need to prioritize three aspects: the precise identification of organ-specific targets and modification sites, as well as the development of clinical translation tools like precision drugs and diagnostic biomarkers. Ultimately, this provides new diagnostic and treatment strategies for complex diseases like comorbidities of the heart-kidney-bone axis.

## Consent for publication

Everyone agrees to publish.

## Avalilability of data and material

Not applicable.

## Ethics approval

Not applicable.

## Funding

This work was supported by the Key Projects of the 10.13039/501100001809National Natural Science Foundation of China (Grant No. 32130052) and 10.13039/501100003787Natural Science Foundation of Hebei Province (CN) - for Yanzhao Young Scientists Project (Grant No. H2023206519) and Tianjin Key Project of Traditional Chinese Medicine (Grant No.2025005) and Tianjin Natural Science Foundation(Grant No.25JCLMJC00290).

## CRediT authorship contribution statement

**Shuangcui Wang:** Software, Writing – original draft. **Maojuan Guo:** Methodology. **Bo Yang:** Visualization. **Tingting Fan:** Investigation. **Xiaojuan Zhang:** Data curation. **Chen Feng:** Investigation. **Yanbin Zhu:** Formal analysis. **Yingze Zhang:** Writing – review & editing. **Wei Chen:** Writing – review & editing.

## Declaration of competing interest

The authors declare that they have no known competing financial interests or personal relationships that could have appeared to influence the work reported in this paper.

## Data Availability

No data was used for the research described in the article.
